# Sexually Transmitted Infections in the Third Age: Rising Risk Among over 65-Year-Old Persons in Southern Sardinia

**DOI:** 10.3390/jcm15207866

**Published:** 2026-10-12

**Authors:** Grazia Vivanet, Federica Perra, Caterina Ferreli, Laura Atzori

**Affiliations:** Dermatology Unit, Department of Medical Sciences and Public Health, University of Cagliari, 09124 Cagliari, Italyatzoril@unica.it (L.A.)

**Keywords:** sexually transmitted infections, older adults, syphilis, condyloma acuminata, sexual health in ageing

## Abstract

**Background/Objectives**: Population ageing and the persistence of sexual activity in later life are reshaping sexual health needs. Older adults are often overlooked in STI surveillance and prevention, and data on infections among those aged ≥65 years remain limited. This study describes the epidemiological and clinical profile of STIs in older adults in Southern Sardinia. Our aim was to describe the epidemiological and clinical characteristics of sexually transmitted infections in adults aged 65 years and older attending a specialist STI outpatient clinic in Southern Sardinia over a 15-year period. **Methods**: We conducted a retrospective longitudinal study of all new STI diagnoses in patients aged ≥65 years attending the Dermatology STI outpatient clinic at the University Hospital of Cagliari between 2010 and 2024. Demographic, behavioural, clinical, and laboratory data were extracted from medical records and analysed descriptively. **Results**: Among 2378 new STI cases, 69 (2.9%) occurred in patients aged ≥65 years (median age 74 years; range 65–83), predominantly men (85.5%) and mostly heterosexual. Most patients reported limited or no condom use, with 84.1% stating that they never used condoms. The most frequent diagnoses were condylomata acuminata (52.2%) and syphilis (31.9%), followed by molluscum contagiosum and other viral and bacterial infections. Relevant co-infections were observed: hepatitis B virus in 17.4%, HIV in 5.8%, and hepatitis C virus in 1.4% of cases. Follow-up ranged from 1 month to 5 years; 46.4% achieved complete clinical resolution, 33.3% required ongoing treatment, and 20.3% were lost to follow-up. **Conclusions**: Although they represent a small proportion of all STI cases, infections in older adults constitute a meaningful and likely underestimated public health issue. Low risk perception, limited condom use, and diagnostic delays contribute to underdiagnosis. Integrating systematic sexual health assessment, counselling, and targeted prevention into geriatric and primary care is essential to improve the early detection and management of STIs in this growing age group.

## 1. Introduction

Population ageing has become one of the major medical issues facing healthcare systems worldwide. Increased life expectancy has led to changing healthcare needs, including more attention to emerging areas such as sexual health. Epidemiological data indicate a growing incidence and prevalence of sexually transmitted infections (STIs) among individuals aged over 60 years [1,2,3,4,5]. Traditionally, sexual health in older people has been largely overlooked in both clinical practice and research. Despite persistent stereotypes suggesting a lack of sexual interest among older adults, evidence demonstrates that many individuals maintain active and satisfying sexual lives [6]. Sexuality remains an important determinant of psychological well-being, interpersonal relationships, and quality of life [7,8,9].

At the same time, there has been a concerning rise in STIs among older adults. Infections such as syphilis, gonorrhoea, genital herpes, HIV, and chlamydia are increasingly diagnosed in older patients. Contributing factors include increased longevity, the widespread use of medications for erectile dysfunction, changing social behaviours, reduced risk perception and inadequate sexual health education. Diagnostic challenges further complicate management as symptoms may be atypical or masked by comorbidities, leading to delayed diagnosis and treatment. In addition, patients’ reluctance to discuss sexual health and limited clinician training in recognising STIs in older adults may contribute to underdiagnosis. This study was designed to identify cases of sexually transmitted infections (STIs) diagnosed in patients aged ≥65 years at the Dermatology Unit of the University Hospital of Cagliari over a 15-year period (2010–2024). Data were analysed to identify epidemiological trends, clinical characteristics, diagnostic approaches and treatment outcomes.

## 2. Materials and Methods

We conducted a retrospective longitudinal observational study to describe disease frequency in relation to variables such as age, sex, and time. Data were collected through a retrospective review of medical records from the Dermatology outpatient clinic dedicated to STIs, which has been part of the Italian sentinel STIs surveillance network since 1991. The inclusion criteria were age ≥ 65 years and a confirmed STI diagnosis established by blood tests, urethral swabs or clinical evaluation. Patients younger than 65 years or diagnosed outside the study period (2010–2024) were excluded. For each patient, the following variables were analysed: year of diagnosis, sex, age, clinical and laboratory diagnosis, sexual orientation, use of barrier methods, presence of sexually transmitted co-infections, duration of follow-up at the STI centre, and clinical outcomes, including cure. Clinical outcome referred to the STI diagnosis managed at the Dermatology STI outpatient clinic and was classified as complete clinical resolution, ongoing treatment/follow-up, or loss to follow-up. Outcomes of concomitant chronic viral infections such as HIV, HBV, and HCV were not included in this classification and were managed or referred according to standard clinical pathways. Previous STI history was assessed during medical history taking and, when a previous infection was reported, patients were asked to provide the corresponding medical or laboratory documentation. Previous STIs were therefore considered documented when supported by available clinical records or test reports. Although the study was retrospective, data collection included detailed medical histories, clinical examination reports and laboratory test results. As part of the sentinel STIs surveillance network, the clinical evaluation includes the collection of data on patients’ sexual history, risk behaviours and knowledge of STIs. Blood samples for HIV, HBV, HCV, VDRL, and TPHA testing were collected by trained healthcare personnel in accordance with standard safety and processing guidelines. Testing for *Neisseria gonorrhoeae* and *Chlamydia trachomatis* was not systematically performed in all patients; specific microbiological testing was carried out only in symptomatic individuals. Data sources were cross-checked against records of cases notified to the Public Health Service in accordance with legal requirements for syphilis, gonorrhoea and scabies.

This study was conducted in accordance with the ethical principles of the 1964 Declaration of Helsinki and its subsequent amendments. Under current Italian legislation, retrospective observational studies of this type do not require formal approval by an ethics committee. Written informed consent was obtained from all participants at the time of their initial enrolment at the STI outpatient clinic, prior to follow-up, and included authorisation for the use of anonymised clinical data for research purposes.

All data retrieved from the medical records were entered into a Microsoft Excel (Microsoft 365, version 2609; Microsoft Corporation, Redmond, WA, USA) database and anonymised using progressive binary coding for subsequent analysis. STI prevalence was calculated as the proportion of positive cases for each infection relative to the total sample.

## 3. Results

We analysed clinical data from 69 patients aged 65–83 years (median age 74 years), representing 2.9% (69/2378) of all new STIs diagnoses recorded between 2010 and 2024. Patients were predominantly male, with 59 men (85.5%) and 10 women (14.5%). Sexual orientation was reported as heterosexual in 81.2% of cases (*n* = 56), homosexual in 2.9% *(n* = 2), and bisexual in 4.3% (*n* = 3), while 11.6% of patients (*n* = 8) did not report their sexual orientation. Regarding condom use, 5.8% of patients (*n* = 4) reported consistent use, 10.1% (*n* = 7) occasional use and the majority, 84.1% (*n* = 58) stated never using condoms. No previous STI was reported or documented in 58 patients (84.1%), while 3 patients (4.3%) had at least one documented previous STI; information on previous STI history was unavailable for 8 patients (11.6%) (Table 1).

The spectrum of diagnosed STIs included several conditions, reflecting heterogeneous clinical presentations within the study population (Table 2).

Regardless of the clinical reason for the initial visit to the STI outpatient clinic, all patients were systematically offered a comprehensive screening panel to detect previously undiagnosed sexually transmitted infections.

Age-stratified analysis showed that 56 patients (81.2%) were aged 65–74 years and 13 (18.8%) were aged 75–84 years, while no patients were aged 85 years or older.

A subset of patients had additional sexually transmitted co-infections, including hepatitis B virus (HBV) in 17.4% of cases (*n* = 12), human immunodeficiency virus (HIV) in 5.8% (*n* = 4), and hepatitis C virus (HCV) in 1.4% (*n* = 1), while the remaining 75.4% (*n* = 52) had no known co-infections.

Follow-up duration ranged from 1 month to 5 years. Clinical outcomes showed that 46.4% of patients (*n* = 32) achieved complete clinical resolution, 33.3% (*n* = 23) remained under ongoing treatment at the Dermatology Unit’s STI outpatient service, and 20.3% (*n* = 14) were lost to follow-up.

## 4. Discussion

The findings of this retrospective single-centre study confirm a non-negligible frequency of STIs among individuals aged over 65 years, accounting for approximately 3% of all new STIs diagnoses at our centre. The population aged ≥ 65 years residing in the Province of Cagliari increased steadily from 74,324 individuals in 2010 to 106,391 in 2024 according to ISTAT data. Over the 15-year observation period, the 69 STI cases recorded at our centre correspond to an estimated average annual rate of approximately 5.1 cases per 100,000 inhabitants aged ≥ 65 years. Although based on a single-centre experience, these results highlight the importance of STIs in the older population, particularly in the context of a steadily growing population potentially at risk, and underscore the need for greater awareness and targeted attention to this phenomenon [10]. These infections are often underestimated in clinical practice and affect a population group for which specific prevention, education and awareness strategies are generally lacking. Older adults are often grouped together with younger or reproductive-age patients, who are traditionally considered to be at higher infectious risk, so reducing attention to the older population.

All data in the present study were collected within the framework of the Italian National Sentinel Surveillance System for Sexually Transmitted Infections [11]. This surveillance network utilizes standardised criteria for data collection and case reporting across participating centres, guaranteeing the quality and consistency of epidemiological information.

The difficulty in focusing on sexual health in older age largely stems from prioritising reproductive and fertility-related aspects—undoubtedly central up to around the age of 50—at the expense of assessing infectious and transmission risks. Contrary to common perceptions, the risk of infection does not disappear with advancing age and may even increase. This is partially due to reduced concern about pregnancy-related prevention after menopause and partially to the persistent misconception that sexual activity in older age is absent or irrelevant. This perceptual bias represents a significant risk factor, as reflected in global reports of increasing HIV diagnoses among individuals aged over 60 years).

The predominance of male patients (85.5%) in our cohort is consistent with the existing literature. Several epidemiological studies indicate that men, particularly older men, are more likely than women of the same age to seek medical attention for sexual dysfunction, potentially increasing the likelihood of STI diagnosis [12,13]. Men more frequently report sexual symptoms and seek care for conditions such as erectile dysfunction or premature ejaculation, whose prevalence increases with age. Similarly, male predominance is well documented in STI clinics, with studies reporting proportions exceeding 80% in European outpatient settings [14]. This pattern is likely to reflect both behavioural factors and a greater tendency among men to seek specialist care [12,13,14,15].

Conversely, the low proportion of female patients (14.5%) may reflect a subgroup of women who, because of cultural or socioeconomic factors, are less likely to access healthcare services for sexual health concerns. However, older women remain at risk of STIs, particularly when exposed to high-risk partners. Gender-specific investigation in this area represents an important field of interest for future research.

A key finding of this study is that 84.1% of patients reported never using condoms. This indicates low awareness of STI risk in older age and exposes this population to potentially serious infections such as syphilis and HIV. This behaviour may be attributable to several factors, including underestimation of risk, lack of targeted sexual health education and generational barriers to adopting protective practices.

**Figure 1:**
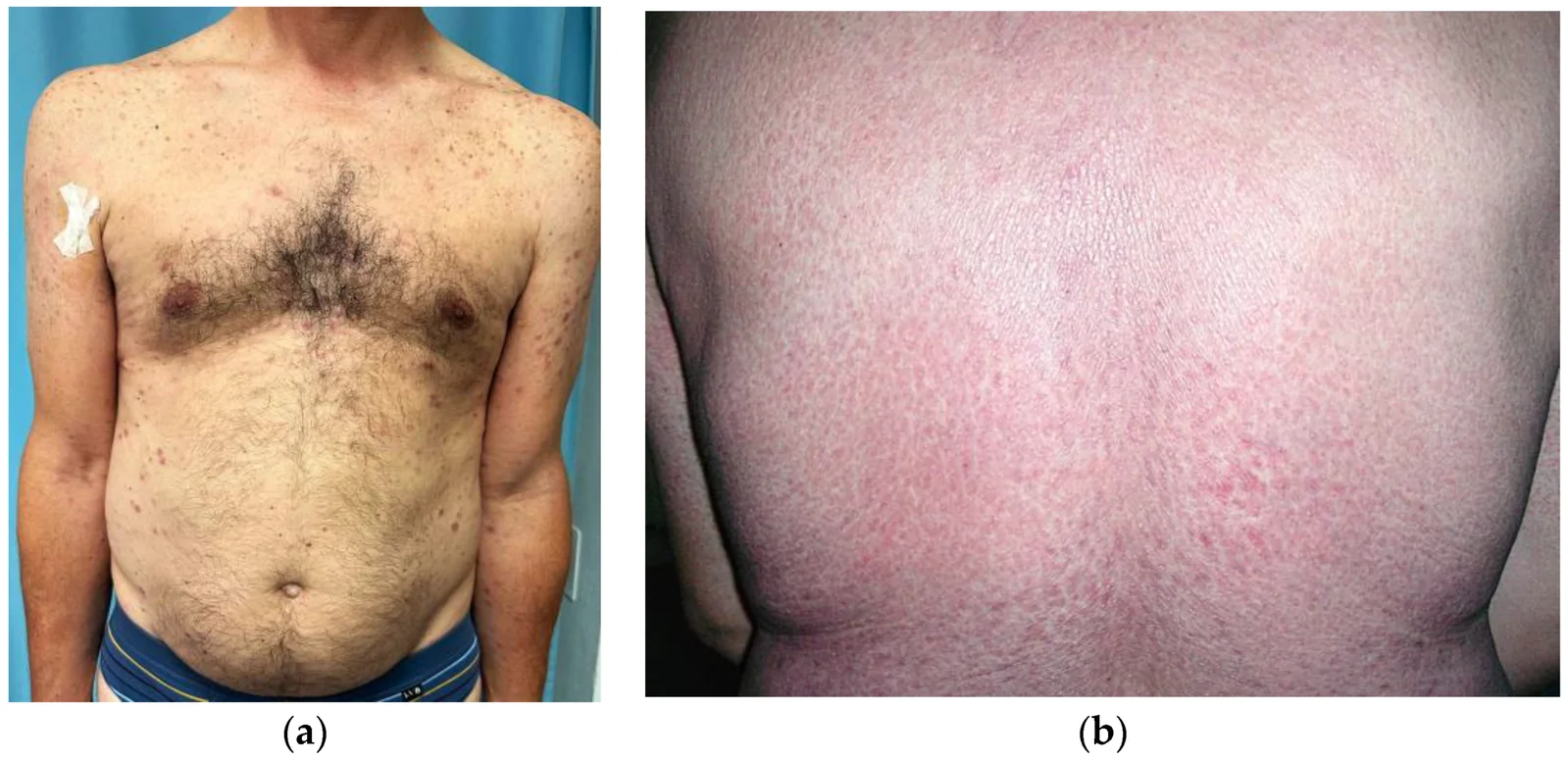
Syphilis. (**a**) Secondary syphilis in an elderly man. (**b**) Secondary syphilis in an elderly woman.

**Figure 2:**
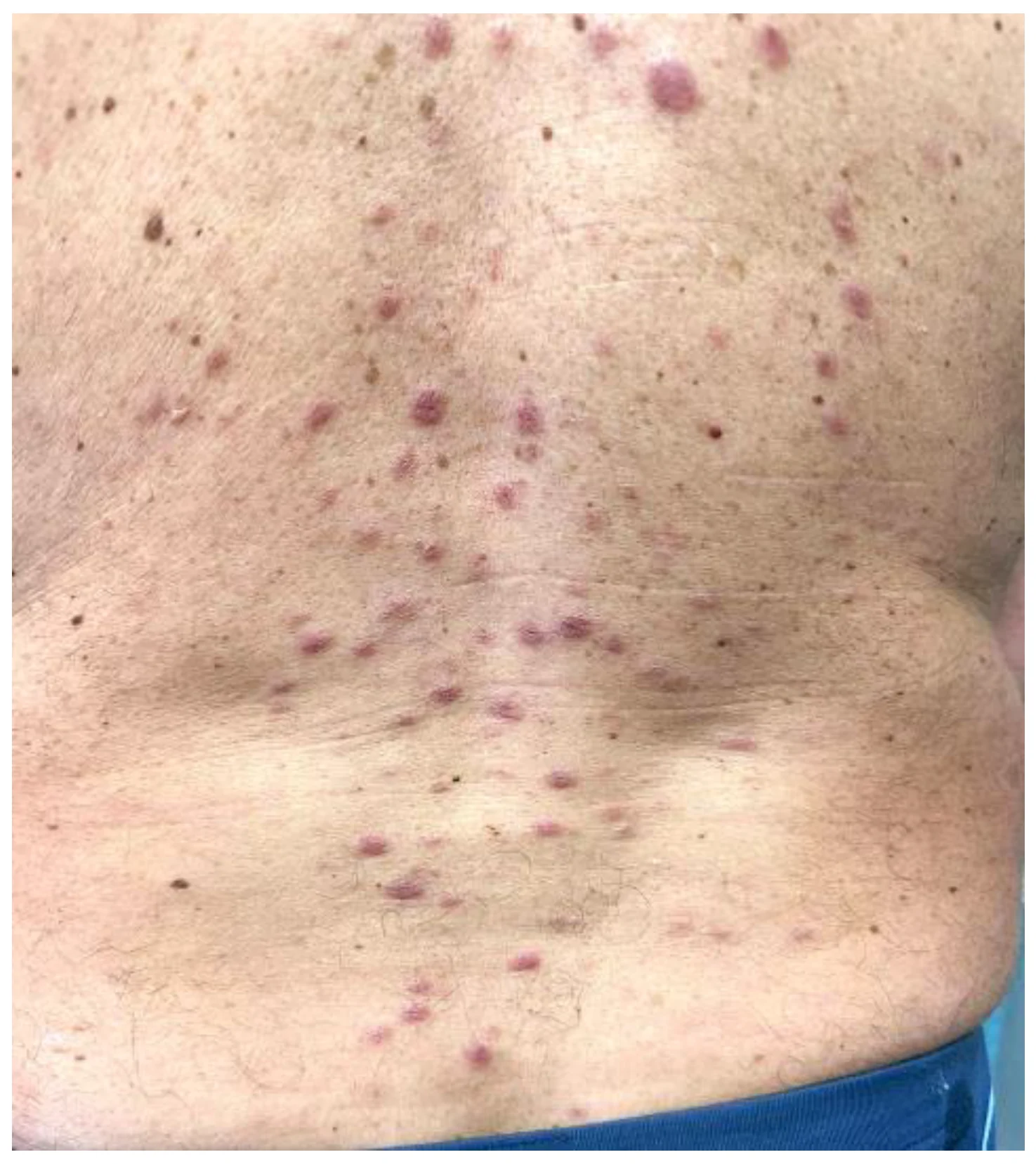
Syphilis, detail. A maculopapular rash in an elderly patient raises a broad differential diagnosis.

The spectrum of diagnoses included a wide range of infections, with a clear predominance of genital warts (52.2%) and syphilis (31.9%) (Table 2). Although syphilis has received increasing attention in recent years, it remains frequently underdiagnosed in older patients, whose symptoms may be mistaken for those of other conditions (Figure 1 and Figure 2). Delayed diagnosis, as with HIV infection, may result in irreversible organ damage affecting the cardiovascular, hepatic, and nervous systems, despite the availability of effective treatment.

This distribution reflects two key epidemiological trends: the persistence of human papillomavirus (HPV) infection and the resurgence of syphilis, particularly among people with high-risk sexual behaviours, including unprotected intercourse and multiple partners [16,17]. The predominance of genital warts suggests that HPV remains an important viral STI among men, favoured by factors such as advanced age, reduced immune function and exposure to new partners. These lesions are associated with recurrence and sometimes progression to anogenital malignancies [17].

Syphilis was the second most frequent diagnosis and may lead to severe neurological and cardiovascular complications if left untreated [16]. The high amount of syphilis observed in our cohort is consistent with the re-emergence of this infection in older adults and highlights the importance of maintaining a high index of clinical suspicion, even in patients presenting with atypical dermatological manifestations [18].

The low prevalence of genital herpes (1.4%), despite its high prevalence in the general population, is likely to reflect underdiagnosis and underreporting. Approximately 70% of individuals infected with HSV-2 are unaware of their condition because of asymptomatic or atypical presentations. In older adults, normalisation of symptoms and low awareness may further contribute to underdiagnosis [19]. Moreover, clinical diagnosis is complicated by symptom overlap with other conditions and the absence of typical lesions at the time of examination [20]. In addition, herpes is not a notifiable disease, and diagnostic tests are often not performed in the absence of evident lesions [21]. The absence of diagnosed gonorrhoea and chlamydial infection in our cohort should be interpreted with caution, as microbiological testing for N. gonorrhoeae and C. trachomatis was not systematically performed in asymptomatic patients. Therefore, asymptomatic infections may have remained undetected, and our findings do not allow us to determine whether the absence of recorded cases reflects age-related biological differences in susceptibility or pathogenicity.

Analysis of co-infections revealed that, although uncommon, their presence is clinically relevant. Rates of HBV (17.4%), HIV (5.8%), and HCV (1.4%) suggest that sexual transmission of viral infections remains possible in older adults, particularly in the context of high-risk partners and unprotected intercourse. These co-infections were identified or documented during specialist evaluation and may have important implications for both individual patients and public health. However, because of limitations within the healthcare system, partner tracing remains limited and may be hindered by privacy concerns.

The detection of co-infections in older men attending STI clinics suggests that high-risk behaviours persist in this population, with prevalence rates exceeding those expected in the general older population. The literature indicates that HBV and HCV infections are increasingly prevalent among older individuals because of cohort effects. Sexual transmission of HBV and HIV is well documented among individuals with multiple partners and those engaging in unprotected intercourse, particularly among men who have sex with men, but also among heterosexual individuals engaging in high-risk behaviours [22,23]. The presence of ulcerative STIs further increases susceptibility to viral transmission.

Another important aspect concerns clinical outcomes and follow-up. While 46.4% of patients achieved clinical resolution, a substantial proportion required prolonged treatment (33.3%), and 20.3% were lost to follow-up. The latter finding is particularly concerning and reflects challenges in long-term management, especially for chronic infections. The reasons for loss to follow-up were not systematically available; therefore, mortality, transfer of care, reduced mobility, stigma, psychological barriers or other causes cannot be distinguished.

The main limitation of this study is its retrospective design, which did not allow assessment of relevant social variables such as living conditions, educational level, or occupational history. Consequently, several potentially important questions remain unanswered.

Nevertheless, the findings demonstrate a substantial gap in both primary and secondary prevention. Limited sexual health education and a lack of targeted screening contribute to continued exposure to infectious risk. It is therefore essential that general practitioners, gynaecologists, and geriatric specialists increase their awareness and actively address sexual health with older patients. Furthermore, healthcare policies should promote education and reduce the stigma surrounding sexuality in later life, through a more inclusive and informed approach among both patients and healthcare professionals.

## 5. Conclusions

This study highlights that STIs represent a relevant and often underestimated issue among older adults. Although their prevalence remains lower than in younger age groups, the findings demonstrate that the risk of infection persists with advancing age and may be influenced by inadequate risk perception and limited use of preventive measures. The low rate of condom use observed in this cohort reflects a lack of targeted sexual health education and awareness, contributing to continued exposure to infection.

Diagnostic challenges, including atypical clinical presentations and overlap with chronic conditions, together with limited communication between patients and healthcare providers, further contribute to underdiagnosis and delayed treatment. In addition, the substantial proportion of patients lost to follow-up underscores the need for improved strategies to ensure continuity of care, taking into account the specific psychosocial and logistical barriers affecting older individuals.

Overall, these findings emphasise the importance of integrating sexual health into routine geriatric care. A more proactive and multidisciplinary approach, combining screening, education, and improved clinical communication, is essential to enhance the prevention, early diagnosis, and management of sexually transmitted infections in this growing segment of the population.

## Figures and Tables

**Table 1:** Demographic, behavioural, clinical, and follow-up characteristics of patients aged ≥65 years diagnosed with sexually transmitted infections.

Characteristic	*n* (%) or Value
**Age, years**	
**Median (range)**	74 (65–83)
65–74 years	56 (81.2%)
75–84 years	13 (18.8%)
≥85 years	0 (0.0%)
**Sex**	
Male	59 (85.5%)
Female	10 (14.5%)
**Sexual orientation**	
Heterosexual	56 (81.2%)
Homosexual	2 (2.9%)
Bisexual	3 (4.3%)
Not reported	8 (11.6%)
**Condom use**	
Consistent	4 (5.8%)
Occasional	7 (10.1%)
Never	58 (84.1%)
**Previous history of STIs**	
No previous STI	58 (84.1%)
≥1 previous STI	3 (4.3%)
Information unavailable/not reported	8 (11.6%)
**Sexually transmitted co-infections**	
HBV infection	12 (17.4%)
HIV infection	4 (5.8%)
HCV infection	1 (1.4%)
No known co-infection	52 (75.4%)
**Follow up duration, range**	1 month–5 years
**Clinical outcome**	
Complete clinical resolution	32 (46.4%)
Ongoing treatment/follow-up	23 (33.3%)
Lost to follow-up	14 (20.3%)

**Table 2:** Distribution of sexually transmitted infection diagnoses in the study population.

Diagnosis	N. of Patients	Percentage (%)
**Condyloma acuminata**	36	52.2%
**Syphilis**	22	31.9%
**Genital candidiasis**	1	1.4%
**Genital herpes**	1	1.4%
**Molluscum contagiosum**	4	5.8%
**Molluscum contagiosum + condyloma acuminata**	1	1.4%
**Non-gonococcal urethritis**	2	2.9%
**Scabies**	2	2.9%

## Data Availability

The data presented in this study are not publicly available due to privacy and ethical restrictions related to patient confidentiality. Anonymized data may be available from the corresponding author upon reasonable request and subject to applicable institutional and legal requirements.

## Abbreviations

The following abbreviations are used in this manuscript:

STI/STIs	Sexually Transmitted Infection(s)
HIV	Human Immunodeficiency Virus
HBV	Hepatitis B Virus
HCV	Hepatitis C Virus
VDRL	Venereal Disease Research Laboratory test
TPHA	Treponema pallidum Hemagglutination Assay
HPV	Human Papillomavirus
HSV-2	Herpes Simplex Virus type 2
ISTAT	Italian National Institute of Statistics/Istituto Nazionale di Statistica
ECDC	European Centre for Disease Prevention and Control
